# Supplementary material for: Chromothripsis during telomere crisis is independent of NHEJ, and consistent with a replicative origin
Source: Genome Res. 2019 May;29(5):737–49. doi: 10.1101/gr.240705.118 (PMC6499312; doi:10.1101/gr.240705.118)
Supplement: Supplemental Material [file supp_gr.240705.118_Supplemental_file_1.zip › contigs/annotated_contigs/DB51/contig.2.DB51_length_448_mean_cov_5.90625.docx]

**DB51_length_448_mean_cov_5.90625**

```
AT|AAACCACCACCATTTGATGGGGAGGATATGCTAGATGATACCTGCTTGCCACCTGTCTTATTTTGGATTCCTCTAAAAGCAGACCC
   >chr20:19494859-19495078 - E=3e-120
TCCGACAAGGATGTGGAGCTATGTGGTTTACTTGGGAGATGACTCCAGGATGCACTGTGCATGAGGGAGGCAGTGCAGCCAGGAAGGGG

AGAAGAGACAAAGACAGTGCTGCTAAGGAGTGAGTGGATGACCT|TACTA|CAAGCCTTATCCAAATTGGTTAAATGTTATCAACTTTG
                                                  >chr6:133568482-133568704 + E=5e-113
TATTTTCTTTGTTTTTGTTTTTTGTTGTTTTAATGCTGGTTTTGAATCTCAAATCTGCACATTTATGTTGAACCAACTAATAAGGCTTG

AAGAGTTAAAGAGTGCATGATGGACTTCTGGAGGCAGGTTTAAATTATAATGGAGCTGCCCATATTTTGGCCCTACATTCAATTTATCT

GGTTAT
```
